# Paeonol Ameliorates Chronic Itch and Spinal Astrocytic Activation via CXCR3 in an Experimental Dry Skin Model in Mice

**DOI:** 10.3389/fphar.2021.805222

**Published:** 2022-01-13

**Authors:** Wen Wang, Qiaoyun Li, Zhongqiu Zhao, Yutong Liu, Yi Wang, Hui Xiong, Zhinan Mei

**Affiliations:** ^1^ School of Pharmaceutical Sciences, South-Central University for Nationalities, Wuhan, China; ^2^ Institute of Ethnomedicine, South-Central University for Nationalities, Wuhan, China; ^3^ Washington University School of Medicine, St. Louis, MO, United States; ^4^ Barnes-Jewish Hospital, St. Louis, MO, United States; ^5^ College of Life Sciences, South-Central University for Nationalities, Wuhan, China

**Keywords:** paeonol, AEW, inflammation, anti-pruritic, CXCR3, astrocyte

## Abstract

Paeonol is a bioactive phenol presents mainly in *Paeonia suffruticosa* Andr. (*Paeoniaceae*), *Paeonia lactiflora* Pall., and *Dioscorea japonica* Thunb. (*Dioscoreaceae*), harboring various pharmacological activities including anti-inflammatory, antioxidant, immune regulatory activity and reverse chemoresistance. Recent reports revealed paeonol exhibited good effects on chronic dermatitis, such as atopic dermatitis (AD) and psoriasis. However, whether paeonol is effective for dry skin disease and its mechanism of action still remain unclear. In this study, we analysed the effects of paeonol on a mouse model of dry skin treated with acetone-ether-water (AEW), which showed impressive activities in reducing scratching behavior and skin inflammation. To elucidate the underlying molecular targets for the anti-pruritic ability of paeonol, we screened the expression of possible chemokine pathways in the spinal cord. The expression of CXCR3 was significantly alleviated by paeonol, which increased greatly in the spinal neurons of AEW mice. In addition, treatment of paeonol significantly inhibited AEW-induced expression of astrocyte activity-dependent genes including *Tlr4*, *Lcn2* and *Hspb1* et al. The inhibitory effects of paeonol on scratching behavior and astrocytic activation in the spinal cord induced by AEW were abolished when CXCR3 was antagonized or genetically ablated. Taken together, our results indicated that paeonol can ameliorate AEW-induced inflammatory response and itching behavior, and reduce the expression of spinal astrocyte activity-dependent genes induced by AEW, which are driven by CXCR3.

## Introduction

With the growth of aging population worldwide, age dependent diseases arouse broad public concern these years, including chronic dermatitis. Xerosis (often called dry skin) is one of the most common dermatological diagnosis in the elderly. The decreased content of natural moisturizing factors and lipids in the stratum corneum causes impaired enzymatic processes leading to the dry skin (White-Chu and Reddy, 2011; Lichterfeld-Kottner et al., 2020). As the most common cause of pruritus in the elderly, dry skin induced repeated scratching in patients resulting in infections, ulcers and other serious complications (Moniaga et al., 2020). In this context, appropriate skin care strategies are effective to maintain and enhance the health and integrity of the elderly’s skin (Lichterfeld-Kottner et al., 2020).

Itch is a unique sensory experience, encoded by genetically distinguishable neurons in the peripheral nervous system (PNS) and central nervous system (CNS) (Dong and Dong, 2018). The itch signals are conveyed from the skin through primary afferents to the spinal dorsal horn (SDH), where it is processed and then sent to the brain via ascending pathways (Koga et al., 2020). Chronic itch has been widely recognized as an important clinical problem, but compared with other sensations (such as pain), its mechanism is poorly understood (Chen and Sun, 2020). Behavioral and pathophysiological evidence from *Trpa1* ^−/−^ mice indicated that the ion channel TRPA1 is required for both transduction of chronic itch signals to the CNS and for the dramatic skin changes triggered by dry-skin-evoked itch and scratching (Wilson et al., 2013; Moniaga et al., 2020). Recently, growing attention has been attracted to the central circuit mechanisms that contribute to itch sensation, including the crucial role of SDH astrocytes (Shiratori-Hayashi et al., 2015; Liu et al., 2016; Liu B. W. et al., 2019; Chen and Sun, 2020).

Chemokines are a family of small secreted proteins that bind to G protein-coupled receptors to trigger intracellular signaling pathways and guide cell migration, proliferation, survival, and inflammation under homeostatic and pathological conditions. Mounting evidence supports the important roles of chemokine signaling in the peripheral and central nervous system in mediating chronic itch (Jiang et al., 2020). For example, the CCL2/CCR2, CXCL12/CXCR4, and CXCL10/CXCR3 signaling of small-diameter sensory neurons are involved in the pathophysiology of allergic contact dermatitis (ACD), eliciting itch- and pain-like behavior in SADBE murine model (Qu et al., 2015; Qu et al., 2017; Jing et al., 2018; Jiang et al., 2019; Su et al., 2020). Administration of a CXCR3 antagonist alleviated the itch behavior in dry skin model mice stimulated with acetone and diethyl ether followed by water (AEW), and *Cxcr3* ^*−/−*^ mice showed attenuated scratching in chronic itch models of dry skin and ACD (Qu et al., 2015; Qu et al., 2017; Jing et al., 2018). Moreover, AEW-induced activation of astrocytes in the spinal cord was suppressed in *Cxcr3* ^*−/−*^ mice (Jing et al., 2018). Thus, targeting specific chemokines or chemokine receptors by natural compounds may provide novel therapeutic potential for the treatment of chronic itch.

Paeonol is a bioactive phenol mainly derived from *Paeonia suffruticosa* Andr. (*Paeoniaceae*), *Paeonia lactiflora* Pall., and *Dioscorea japonica* Thunb. (*Dioscoreaceae*) (Adki and Kulkarni, 2020), which have been applied for various diseases including fever, headache, cold, inflammation, skin diseases, neuralgia, allergy, rheumatoid arthritis, etc (Zhang et al., 2019). Since the first pharmacological activity of paeonol was reported in Traditional Chinese Medicine (TCM) 50 years ago (Harada and Yamashita, 1969), mounting evidence have reported the numerous pharmacologic effects of paeonol, such as anti-oxidant, anti-inflammatory, anti-cancer, apoptosis-inducing and neuroprotective activities (Jin et al., 2016; Liu et al., 2018; Gao et al., 2019; Zhang et al., 2020; Adki and Kulkarni, 2021). Recently, paeonol has been exhibited as a potential therapeutic strategy for the treatment of allergic inflammatory conditions in psoriasis and atopic dermatitis (AD) (Meng et al., 2017; Meng et al., 2019), but whether paeonol is effective in the treatment of dry skin? And if yes, what is the possible mechanism? In order to answer these questions, we carried out the experimental work of this study.

## Materials and Methods

### Reagents

Paeonol (Cat# 552-41-0, ≥98% purity) was purchased from Sichuan Weikeqi Biological Technology Co., Ltd (Sichuan, China) and made into 5% paeonol ointment (the approximate final volumetric molar concentration of paeonol is 300 mM) with other ingredients consist of stearic acid, potassium carbonate, glyceryl monostearate, triethanolamine, glycerin and appropriate amount of water. The positive control drug TRPA1 antagonist HC-030031 (Cat# HY-15064, ≥95.91% purity) and CXCR3 antagonist AMG487 (Cat# HY-15319, ≥99.51% purity) were purchased from MedChemExpress (United States).

### Animals

Male C57BL/6J mice of 7 weeks (±20 g) were purchased from Liaoning Changsheng Biotechnology Co., Ltd (Changchun, China). *Cxcr3* ^*−/−*^ mice (Jing et al., 2018) were provided by Dr. Yongjing Gao at Nantong University in China. All mice were kept in the condition of 22 ± 2°C with 55 ± 15% humidity under a 12 h light/dark cycle and free access to food and water in air-filtered cages. All animal procedures were performed in accordance with the Guidelines for Care and Use of Laboratory Animals of South-Central University for Nationalities (SYXK 2016-0089), and were approved by the Animal Ethics Committee of South-Central University for Nationalities (2017-SCUEC-ACE-023).

### Mouse Model of AEW and Treatment

The AEW model was chosen for *in vivo* pharmacological study of dry skin and established as previously described (Wilson et al., 2013; Zhao et al., 2013; Moniaga et al., 2020). Briefly, the nape of mice was shaved 2 days in advance. Dry skin was induced twice a day for six consecutive days by application of a 1.5 ml 1:1 mixture of acetone and diethyl ether for 20 s, followed by clean water for 30 s, and the animals of blank group were treated with water only. All AEW-induced mice were randomly divided as follows (*n* = 6–7 each group): Model group (applied with Blank matix ointment, referred to as the “AEW” group), low-dose group (applied with Pae ointment, 50 mg/kg, referred to as “Pae-L” group), middle-dose group (applied with Pae ointment, 100 mg/kg, referred to as “Pae-M” group), high-dose group (apply with Pae ointment, 150 mg/kg, referred to as “Pae-H” group), and positive control group (intraperitoneal injection with TRPA1 inhibitor HC-030031 (Fernandes et al., 2013; Oh et al., 2013; Patricio et al., 2015; Wilzopolski et al., 2021), 10 mg/kg, referred to as “PC” group). We refer to previous studies to determine the *in vivo* dose of paeonol (Xue et al., 2017; Xiao et al., 2020). The drugs were administrated once daily at 12:00 AM from day 4 to day 6. On day 7, the spontaneous scratching behavior of mice was recorded for 1.5 h. For behavioral test using CXCR3 antagonist, mice were pre-treated with AMG487 (5 mg/kg) 30 min before paeonol application (150 mg/kg) on day 7 (*n* = 7–8 mice per group). The hind paw scratching behaviors were recorded for 60 min and analyzed by people blinded to the experimental design, which is defined as a lifting of the hind limb towards the shaved area at the back of the neck and then a replacing of the limb back to the floor (Sun et al., 2009; Wang et al., 2020).

### Pathomorphological Study of Skin

The nape skin of mice was collected and fixed in 4% paraformaldehyde (PFA), routinely dehydrated, embedded in paraffin, and sectioned. Haematoxylin and eosin (H&E) and toluidine blue staining were performed as previously described (Zhao et al., 2013; Xiang et al., 2020). After the stained sections were dehydrated, cleared and sealed, they were visualized under a microscope for photograph. ImageJ software was used to measure the thickness of epidermis and count the number of mast cells of dermis (*n* = 3 mice per group).

### ELISA Assay

Protein levels of IL-1β, IL-4 and IL-6 in serum were measured using ELISA kits (Bio-Swamp Life Science, Wuhan, China) according to the manufacturer’s protocol (*n* = 4–8 each group). The absorbance was measured at 450 nm using a microplate reader (Thermo Scientific, Juensuu, Finland) (Wang et al., 2020).

### Real-Time Quantitative PCR

RT-qPCR was performed as previously described (Feng et al., 2017). Briefly, total RNA of spinal cord was extracted using RNAiso Plus (Takara) and reverse transcribed using a Revert Aid First-Strand cDNA Synthesis kit (Thermo) according to the manufacturer’s instructions. The cDNA was subjected to RT-qPCR with corresponding primer sets using SYBR Green Master Mix (Vazyme Biotech Co. Ltd., Nanjing, China) (Liu S. et al., 2019; Wang et al., 2020), and the reaction was hot-started at 95°C for 2 min and then incubated at 95°C for 15 s, 60°C for 30 s, and 72°C for 1 min for 40 cycles. The primer sequences of targeted genes are listed in Table 1.

**TABLE 1:** Oligo nucleotide sequences of the primers used for qPCR.

Gene	Forward (5′ to 3′)	Reverse (5′ to 3′)
*Actin*	GTA​CTC​TGT​GTG​GAT​CGG​TGG	AAA​CGC​AGC​TCA​GTA​ACA​GTC​C
*Ccl1*	GCA​TGC​TTA​CGG​TCT​CCA​ATA​G	GCA​GGG​GTT​CAC​CTT​CTT​CA
*Ccl2*	GAT​GCA​GTT​AAC​GCC​CCA​CT	ACC​CAT​TCC​TTC​TTG​GGG​TC
*Ccl4*	GCT​GTT​TCT​CTT​ACA​CCT​CCC​G	CAG​TTC​AAC​TCC​AAG​TCA​CTC​ATG​T
*Ccl5*	TGC​CCA​CGT​CAA​GGA​GTA​TTT	GAT​GTA​TTC​TTG​AAC​CCA​CTT​CTT​C
*Ccl6*	TAT​CCT​TGT​GGC​TGT​CCT​TGG	TGA​TAA​AGA​TGA​TGC​CCG​GCT
*Ccl7*	TTC​TGT​GCC​TGC​TGC​TCA​TAG	CTT​CCA​TGC​CCT​TCT​TTG​TCT
*Ccl8*	CTG​CTC​ATA​GCT​GTC​CCT​GTC​A	CAC​TGG​ATA​TTG​TTG​ATT​CTC​TCG​T
*Ccl9*	TGG​GTC​TGC​CCA​CTA​AGA​AG	CCC​TTG​CTG​TGC​CTT​CAG​AC
*Ccl11*	CTG​CTC​ACG​GTC​ACT​TCC​TTC	GAA​GAC​TAT​GGC​TTT​CAG​GGT​GC
*Ccl12*	GCT​ACC​ACC​ATC​AGT​CCT​CAG​G	TGG​CTG​CTT​GTG​ATT​CTC​CTG​T
*Ccl17*	CGA​GAG​TGC​TGC​CTG​GAT​TAC	CCC​TGG​ACA​GTC​AGA​AAC​ACG
*Ccl20*	CCT​TCC​AGA​GCT​ATT​GTG​GGT​T	CTC​TTA​GGC​TGA​GGA​GGT​TCA​C
*Ccl2*	CTC​AAA​ATC​CTG​CCG​CAA​GC	AGG​TGA​GTA​AAG​GTG​GCG​TC
*Cxcl1*	CCA​AAC​CGA​AGT​CAT​AGC​CA	TGG​GGA​CAC​CTT​TTA​GCA​TCT
*Cxcl2*	GCT​GTC​CCT​CAA​CGG​AAG​AA	CAG​GTA​CGA​TCC​AGG​CTT​CC
*Cxcl5*	CAC​TCG​CAG​TGG​AAA​GAA​CG	CGT​GGG​TGG​AGA​GAA​TCA​GC
*Cxcl9*	TCC​CCT​AAA​TCT​TCC​ACA​GTG​C	AGG​CCA​AAG​GTT​AGT​TAG​GCA​A
*Cxcl10*	AAA​TCA​TCC​CTG​CGA​GCC​TAT​C	CTA​GCC​ATC​CAC​TGG​GTA​AAG​G
*Cxcl11*	GGC​TTC​CTT​ATG​TTC​AAA​CAG​GG	CAC​TTC​AAC​TTT​GTC​GCA​GCC
*Cxcl13*	TAT​GTG​TGA​ATC​CTC​GTG​CCA​A	GCT​TCA​GGC​AGC​TCT​TCT​CTT​A
*Ccr1*	AGG​TTG​GGA​CCT​TGA​ACC​TT	TGG​AGT​GGA​GTC​CCC​ATA​GT
*Ccr2*	AGG​AGC​CAT​ACC​TGT​AAA​TGC	TAG​TCA​TAC​GGT​GTG​GTG​GC
*Ccr3*	AAC​TTG​CAA​AAC​CTG​AGA​AGC	GGG​TGG​TGC​CCA​CTC​ATA​TT
*Ccr5*	AGC​CGG​GAA​GGT​AGT​CTC​AT	AGT​CCC​GGT​GTG​GTA​GGA​TT
*Ccr6*	CTG​GGC​AGT​TAC​TCA​TGC​CA	CAC​TGC​CAC​ACA​GAT​GAC​CT
*Ccr7*	GGC​CAA​CTT​CAA​CAT​CAC​CAA​T	GCA​TAC​AAG​AAA​GGG​TTG​ACG​C
*Ccr8*	GTG​GGC​AGC​TCT​GAA​ACC​TC	GAG​GAG​GAA​CTC​TGC​GTC​AC
*Ccr9*	GCA​GGC​TGT​TGA​CGC​TTA​TG	CCT​TCG​GAA​TCT​CTC​GCC​AA
*Ccr10*	ATG​TCC​AGG​CTT​TCA​GTC​GG	GAG​GTG​GGA​GAT​CGG​GTA​GT
*Cxcr2*	GAG​CCA​CTC​TGC​TCA​CAA​AC	AGC​AGA​GTC​ACC​AGG​ACG​TA
*Cxcr3*	AGC​CAT​GTA​CCT​TGA​GGT​TAG​T	AGG​TTC​TGT​CAA​AGT​TCA​GGC​T
*Cxcr4*	TGG​AAC​CGA​TCA​GTG​TGA​GT	TGT​CCG​TCA​TGC​TCC​TTA​GC
*Cxcr5*	CAG​CAC​AAA​CCT​TCT​CGA​CAT​C	GCT​GTT​ACT​GTA​GAA​GGC​CAG​T
*Cx3cr1*	CAC​TTG​CCT​CTG​GTG​GAG​TC	GGA​AGG​AGG​TGG​ACA​TGG​TG
*Tlr4*	TGG​CTG​GTT​TAC​ACG​TCC​AT	TGC​AGA​AAC​ATT​CGC​CAA​GC
*Lcn2*	TGA​GTG​TCA​TGT​GTC​TGG​GC	GAA​CTG​ATC​GCT​CCG​GAA​GT
*Hspb1*	ATC​ACT​GGC​AAG​CAC​GAA​GA	GGC​CTC​GAA​AGT​AAC​CGG​AA
*Cd44*	CCT​CTG​CCA​GGC​TTT​CAA​CA	TGC​ACA​GAT​AGC​GTT​GGG​AT
*Cp*	GGT​CCT​GTC​ATT​TGG​GCA​GA	CAT​GGG​AGG​CTT​GCT​GTG​A
*Serpina3n*	TGG​CCT​CCA​TCA​ACA​CTG​AC	AAA​GCC​CTG​GTG​GAT​GTC​TG
*Vim*	GCC​AGC​AGT​ATG​AAA​GCG​TG	ACC​TGT​CTC​CGG​TAC​TCG​TT
*Gfap*	GGC​GAA​GAA​AAC​CGC​ATC​AC	GGT​GAG​CCT​GTA​TTG​GGA​CAA

### Western Blotting

The cervical spinal cord of mice was homogenized in the ice-cold RIPA lysis buffer (Bimake, China) containing protease inhibitors (Bimake, China) and phosphatase inhibitors (Roche, Germany) (Xu et al., 2021), lysed for 30 min, and then centrifuged at 12,000 rpm 4°C for 10 min. Next, the supernatant was mixed with 5× SDS loading buffer and incubated at 95°C for 10 min. Then, the boiled protein lysate was separated by 10% SDS-PAGE and transferred to 0.22 μm PVDF membranes. After blocking with 5% BSA for 2 h at room temperature (RT), the membranes were incubated with primary antibodies at 4°C overnight, washed in TBST, and incubated with appropriate HRP-conjugated secondary antibodies for 2 h at RT. The protein bands were visualized by using BeyoECL Plus (P0018, Beyotime, China), and a densitometry analysis was performed on an imaging system (Tanon Science and Technology Co., Ltd., Shanghai, China) (Liu S. et al., 2019). The primary antibodies used in this study are anti-CXCR3 (26756-1-AP, Proteintech, 1:1,000) and anti-GAPDH (#6176106, CST, 1:10,000).

### Immunofluorescence Staining

Mice was perfused with 4% PFA and the cervical spinal cord was dissected. Next, the tissues were fixed in 4% PFA overnight at 4°C, followed by processing in 20% sucrose, embedded in OCT, and then sectioned into 20 μm, and subjected to tissue staining after cryosection. A blocking solution was prepared with 0.1% Tritox-100, 3% goat serum, 0.1% BSA plus PBS solution, and blocked at room temperature for 1 h (Xiang et al., 2020). After blocking, sections were incubated overnight at RT with the specific primary antibodies (anti-CXCR3, 1:5,000, 26756-1-AP, Proteintech; anti-NeuN, 1:200, Millipore, MAB377; anti-GFAP, 1:500, Millipore, MAB360; anti-CD11b, 1:200, Abcam, ab8878). Then, they were washed 3 times in PBS, and incubated with the appropriate secondary antibodies (Alex Fluor™ 647 goat anti-rabbit IgG (H + L), 1:1,000, Thermo Fisher Scientific, #1851447; Alex Fluor™ 568 goat anti-mouse IgG (H + L), 1:300, Thermo Fisher Scientific, 1862187) for 1 h at RT. After incubated with 5 μg DAPI, tissue sections were washed, mounted and then imaged after drying. All imaging were performed on a Zeiss Axio Observer 7 Fluorescence Microscope. The immune signal intensity was quantified in a semi-automatic manner using Image-Pro Plus software and 4–5 images were counted for each mouse as described previously (Zhao et al., 2013; Xiang et al., 2020).

### Statistical Analysis

All data are presented as the mean ± SEM. GraphPad Prism five software (San Diego, California, United States) was used for the statistical analysis of the data and generation of the graphics. Student’s t test was used to analyse statistical comparisons between two groups. One-way analysis of variance (ANOVA) followed by Bonferroni’s post hoc test was used for multiple group comparisons (Liu S. et al., 2019). *p* value < 0.05 was considered statistically significant.

## Results

### Paeonol Attenuated the Scratching Behavior in AEW-Induced Mice

**FIGURE 1:**
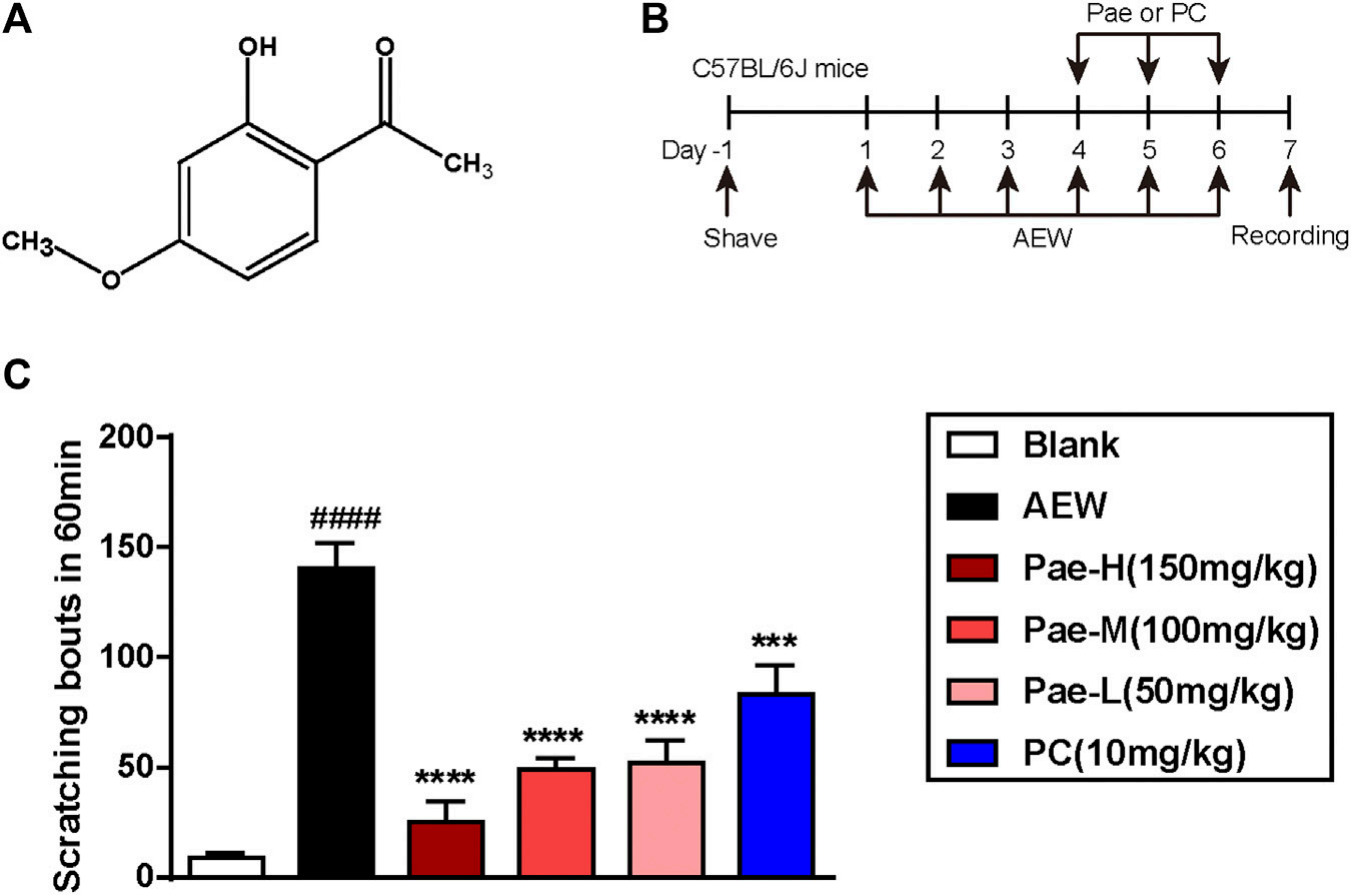
Paeonol alleviated AEW-induced scratching behavior of dry skin mice. **(A)** The chemical structure of paeonol. **(B)** Schematic diagram of the animal experimental protocol. Spontaneous scratching of mice was videotaped and analysed. **(C)** The behavior results of different groups on day 7. Data were expressed as mean ± SEM. ^###^ *p* < 0.001, compared with the blank group. ^**^ *p* < 0.01, ^***^ *p* < 0.001, compared with the AEW model (*n* = 6–7 each group). Pae-H, paeonol-high dose; Pae-M, paeonol-middle dose; Pae-L, paeonol-low dose; PC, positive control drug, HC-030031. The relevant labels in the following figures are the same unless otherwise mentioned.

Paeonol (2-hydroxy-4-methoxyacetophenone) is a bioactive phenol (Figure 1A) which has various pharmacological potential for clinical therapeutics (Jin et al., 2016; Meng et al., 2017; Liu et al., 2018; Gao et al., 2019; Meng et al., 2019; Adki and Kulkarni, 2020). To access the effect of paeonol against dry skin, mice were applied with AEW on the shaved nape twice daily for 6 days, and paeonol or positive control (PC) drug were administrated for 3 days starting from day 4 (Figure 1B). Since TRPA1 is required for both transduction of chronic itch signals to the CNS and for the dramatic skin changes triggered by dry-skin-evoked itch and scratching (Wilson et al., 2013; Feng et al., 2017), we chose its antagonist HC-030031 as the PC. Consistently, mice stimulated with AEW exerted intense spontaneous scratching on day 7 (Figure 1C), and three doses of paeonol significantly reduced the scratching bouts in a dose-dependent manner. It’s worth noting that paeonol exhibited a trend to be superior to PC in alleviating the chronic itch induced by AEW.

### Paeonol Improved the Pathologic Changes of Skin in Mice Induced by AEW

**FIGURE 2:**
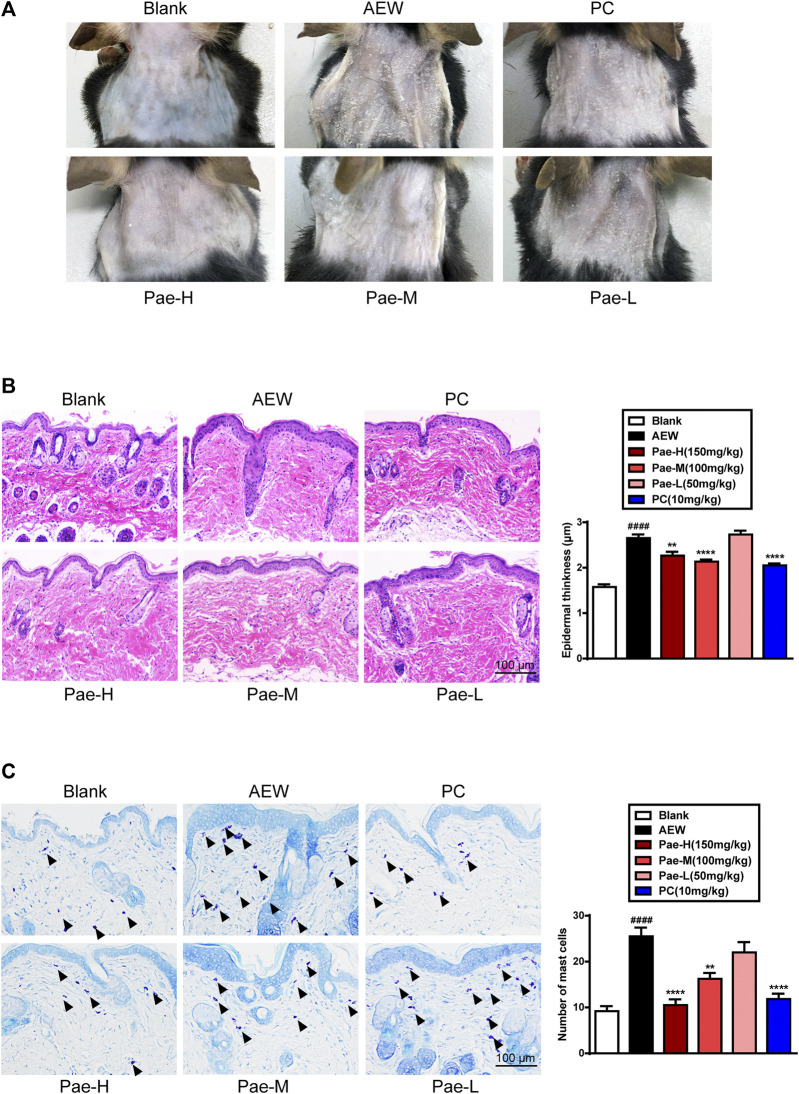
Paeonol improved the pathological changes of dorsal neck skin in AEW mice. **(A)** The representative images of dorsal neck skin of each group on day 7. **(B)** The representative images of Haematoxylin and eosin (H&E) staining and the thickness of epidermis were measured. **(C)** The representative images of toluidine blue staining and the mast cell number were counted. Data were expressed as mean ± SEM. ^###^ *p* < 0.001, compared with the blank group; ^**^ *p* < 0.01, ^***^ *p* < 0.001, compared with the AEW model group (*n* = 3 each group).

As described in previous studies, the mice neck stimulated with AEW showed severe skin lesions characterized by a dry, scaly and rough surface (Zhao et al., 2013). Administration of paeonol (150 mg/kg, 100 mg/kg) efficiently improved these pathologic changes, while the skin appearance of PC group showed no significant difference with that of model mice (Figure 2A). To further explore the anti-inflammatory effect of paeonol, pathological analysis of skin slices were conducted. Results of H&E (Figure 2B) and toluidine blue (Figure 2C) staining indicated that paeonol (150 mg/kg, 100 mg/kg) showed good effects on improving the corneous hyperplasia and mast cell infiltration induced by AEW, which was similar to PC drug.

### Paeonol Decreased the Pro-Inflammatory Cytokine Levels in Serum of AEW Mice

**FIGURE 3:**
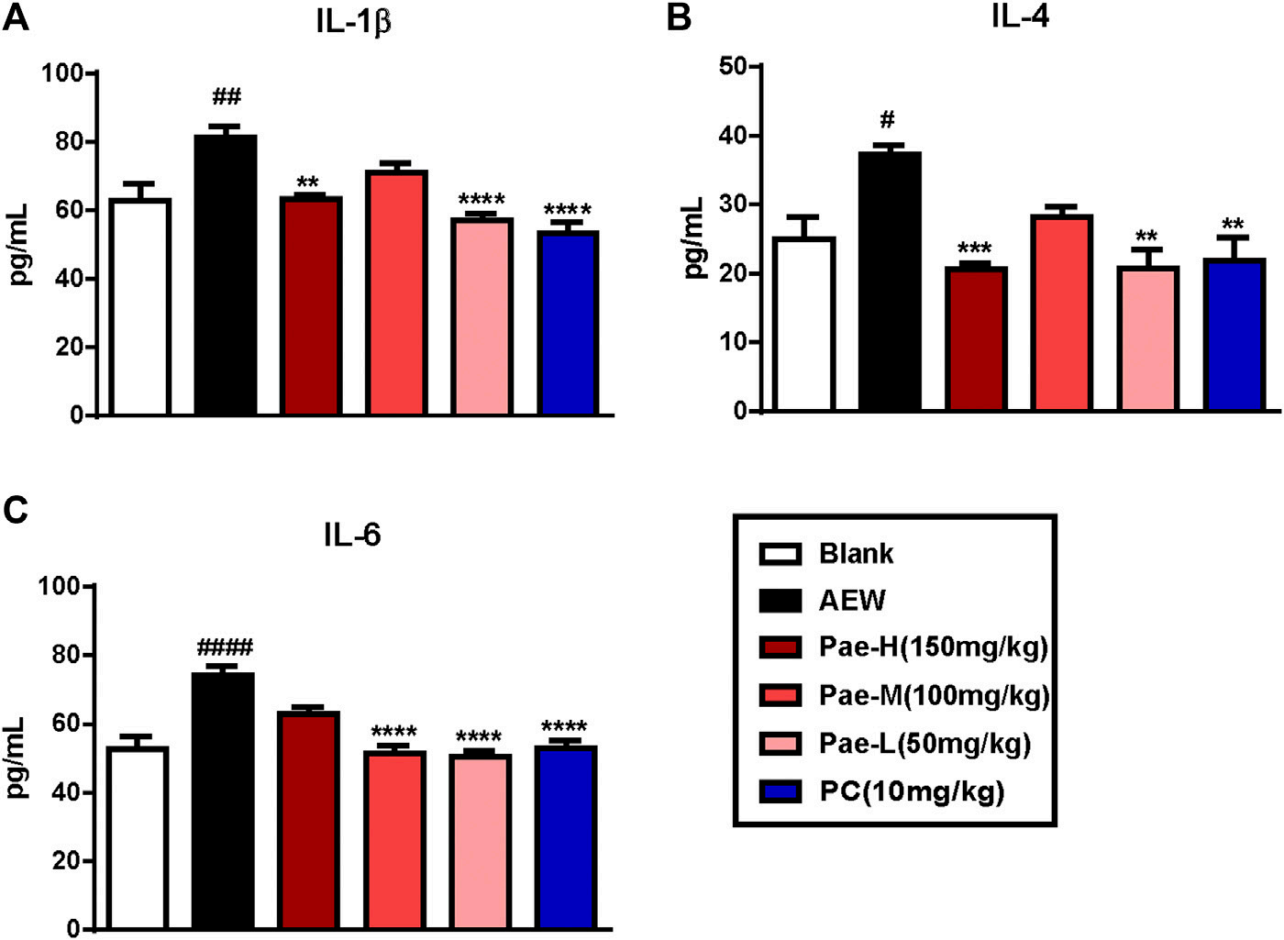
Paeonol reduced protein levels of pro-inflammatory cytokines in the serum of AEW mice. **(A–C)** Administration of paeonol decreased the levels of IL-1β **(A)**, IL-4 **(B)**, IL-6 **(C)** in the serum of AEW-induced mice. Data were expressed as mean ± SEM. ^#^ *p* < 0.05, ^##^ *p* < 0.01, ^####^ *p* < 0.0001, compared to the blank group; ^∗∗^ *p* < 0.01, ^∗∗∗^ *p* < 0.001, ^∗∗∗^ *p* < 0.0001, compared to the AEW model group (*n* = 4–7 each group).

Alterations in the protein levels of cytokines IL1-β, IL-4 and IL-6 were analyzed by ELISA in order to illustrate the anti-inflammatory effects of paeonol. The expression of IL-1β, IL-4 and IL-6 were increased in the serum of AEW group compared to the blank group (Figures 3A–C). On the other hand, treatment with paeonol inhibited AEW-induced cytokine expression, and the protein levels of IL-1β, IL-4 and IL-6 were significantly lessened in paeonol-treated mice.

### Paeonol Reduced the Upregulation of CXCR3 in the Spinal Cord of AEW Mice

**FIGURE 4:**
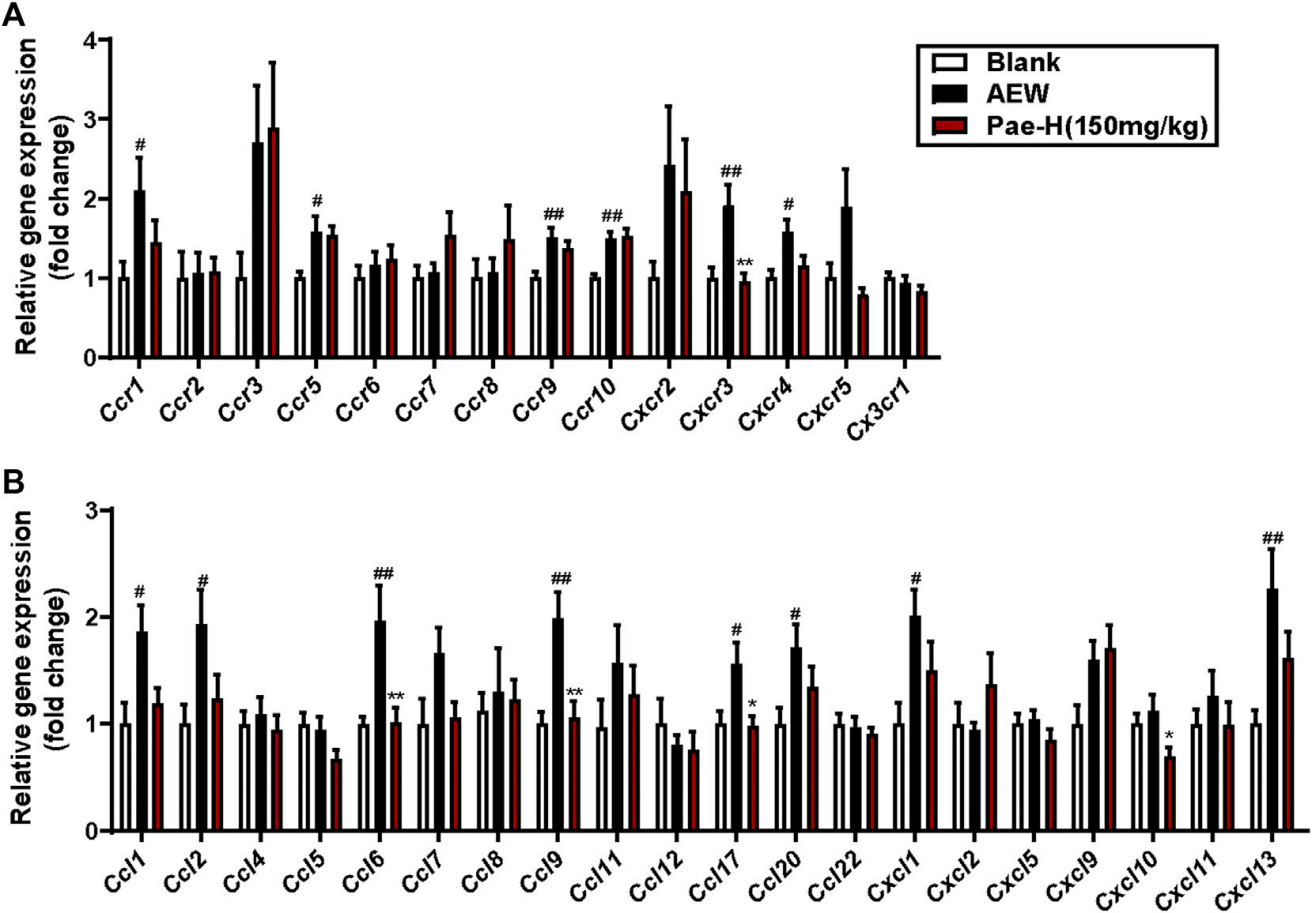
The effects of paeonol on the expression of chemokine receptors and ligands in the spinal cords. The increased mRNA levels of chemokine receptor *Cxcr3* **(A)** and chemokines including *Ccl6*, *Ccl9*, and *Ccl17* **(B)** in the spinal cord of AEW mice were decreased by paeonol application. Data were expressed as mean ± SEM. ^#^ *p* < 0.05, ^##^ *p* < 0.01, ^###^ *p* < 0.001, compared to the blank group; ^∗^ *p* < 0.05, ^∗∗^ *p* < 0.01, compared to the AEW model group (*n* = 4–8 each group).

The chemokine signalling of CNS plays a vital role in mediating chronic itch (Jing et al., 2018; Jiang et al., 2020). Thus, we used RT-qPCR to screen the expression of spinal chemokine pathways to explore the potential mechanism for paeonol’s anti-pruritic activity. Among chemokine receptors (Figure 4A) and chemokine ligands (Figure 4B) increased in the spinal cord of AEW group, paeonol significantly reduced the expression of CXCR3. Besides, paeonol also reduced the mRNA level of CXCL10, which is the one of the ligands of CXCR3. Emerging evidence proposed that CXCR3/CXCL10 signaling in the spinal cord may mediate central sensitization underlying chronic itch and alloknesis in AEW model (Jing et al., 2018). Therefore, we proposed that paeonol may exhibited an anti-pruritic effect via suppressing CXCR3 in the spinal cord.

### The Expression Profile of Chemokine Receptor CXCR3 in the Spinal Cord of AEW Mice

**FIGURE 5:**
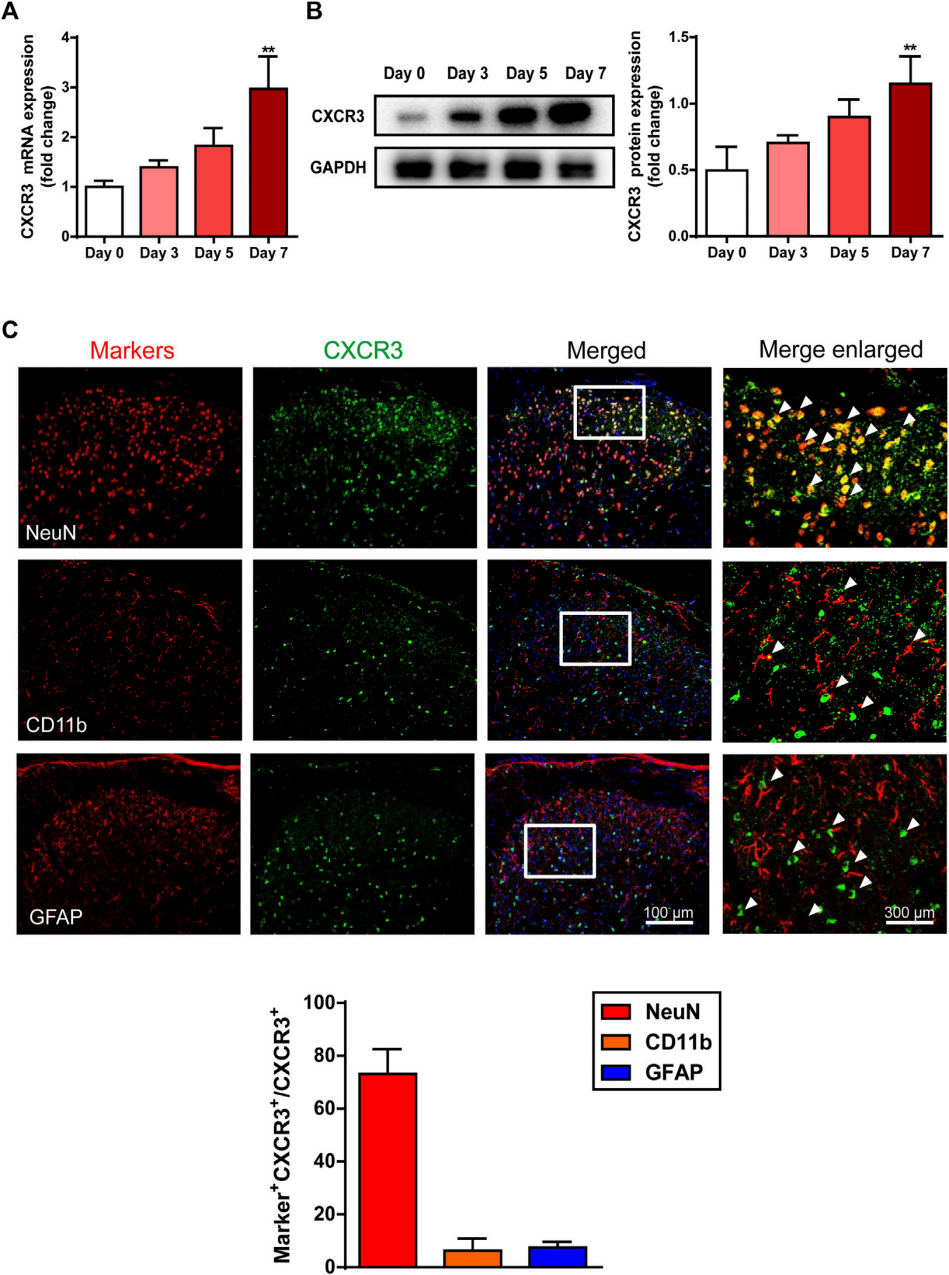
The expression profile of CXCR3 in the spinal cord in the AEW-stimulated mice. The mRNA **(A)** and protein **(B)** level of CXCR3 in the spinal cord increased as stimulation of AEW went on (*n* = 3–7 each group). **(C)** Immunofluorescence co-staining images of CXCR3 with neuron marker (NeuN), microglial marker (CD11b) and astrocyte marker (GFAP) in the spinal dorsal horn of AEW mice (*n* = 4 each group). Data were expressed as mean ± SEM. ^∗∗^ *p* < 0.01, compared to the Day 0 group.

To further verify the contribution of CXCR3 to AEW-induced pruritus of mice, we examined the expression of spinal CXCR3 at a series of time points. Results of RT-qPCR and western blotting demonstrated that both mRNA and protein levels of CXCR3 gradually elevated in the spinal cord of AEW mice (Figures 5A,B), suggesting that CXCR3 was likely involved in the pathogenesis of dry skin. Then, the expression profile of CXCR3 in the spinal dorsal horn was investigated by immunofluorescence (IF) assay. Double-staining of CXCR3 with NeuN, CD11b and GFAP respectively showed that CXCR3 dominantly located in NeuN^+^ neurons, but not CD11b^+^ microglia cells or GFAP^+^ astrocytes (Figure 5C) in the spinal dorsal horn.

### Paeonol Inhibited the Expression of Neuronal CXCR3 in the Spinal Cord of AEW Mice

**FIGURE 6:**
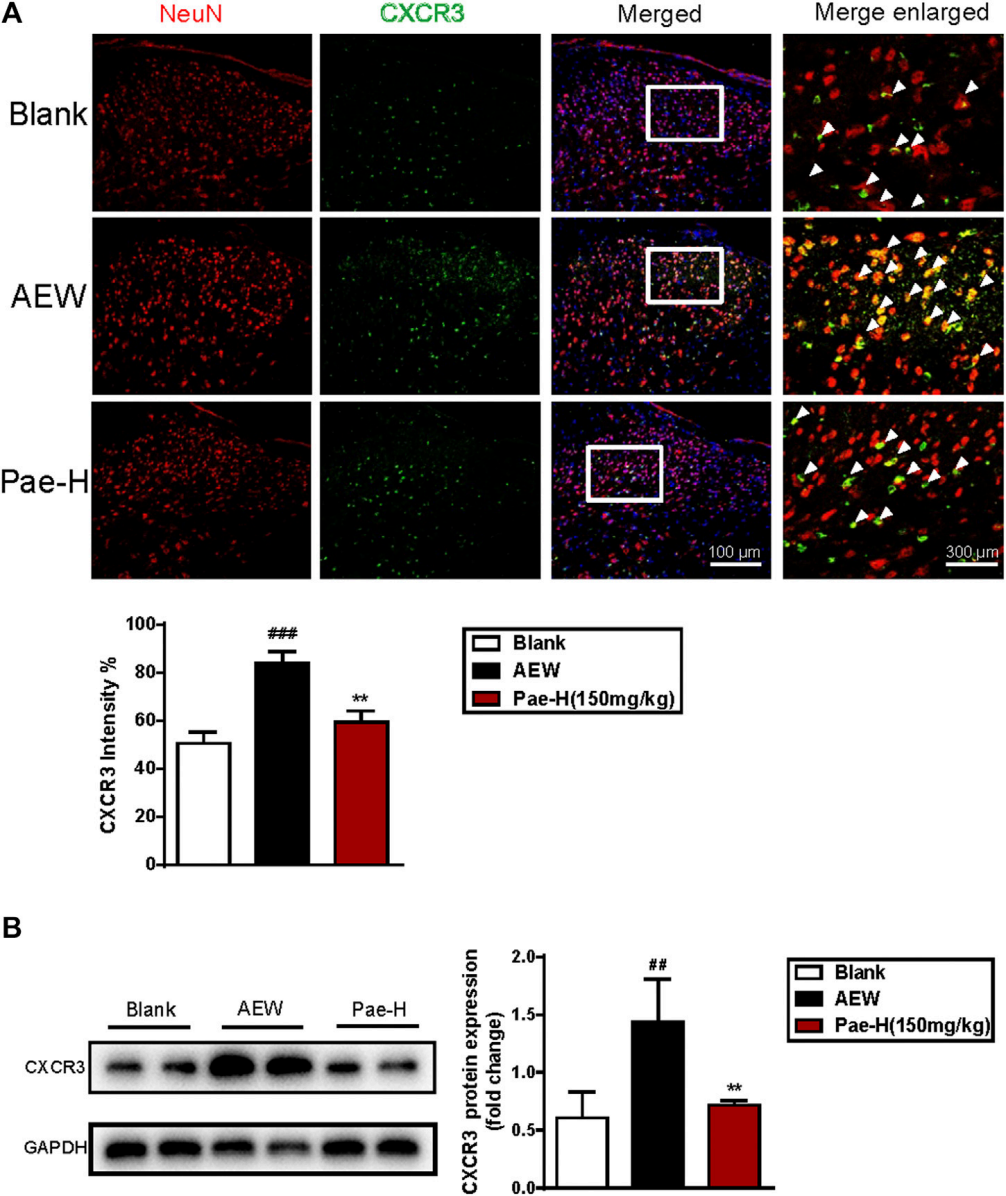
Paeonol decreased the expression of CXCR3 in the spinal neurons of AEW group. **(A)** The results of immunofluorescence indicated paeonol downregulated the CXCR3^+^NeuN^+^ double-positive cell ratio in the AEW spinal cord (*n* = 4 each group). **(B)** The results of western blot demonstrated paeonol reduced the protein level of spinal CXCR3 in AEW model group (*n* = 4 each group). Data were expressed as mean ± SEM. ^##^ *p* < 0.01, ^###^ *p* < 0.001, compared to the blank group; ^∗∗^ *p* < 0.01, compared to the AEW model group.

Next, we analyzed the effects of paeonol on CXCR3 expression of spinal neurons by IF staining. As shown in Figure 6A, AEW stimulation dramatically raised the ratio of CXCR3^+^NeuN^+^ double positive cells in the spinal dorsal horn, and application of paeonol efficiently reversed the increased intensity of CXCR3 in NeuN^+^ neurons. Results of western blotting also supported this consequence (Figure 6B), as AEW-induced upregulation of spinal CXCR3 was significantly decreased by paeonol administration.

### Paeonol Suppressed AEW-Induced Chronic Itch in a CXCR3-Dependent Way

**FIGURE 7:**
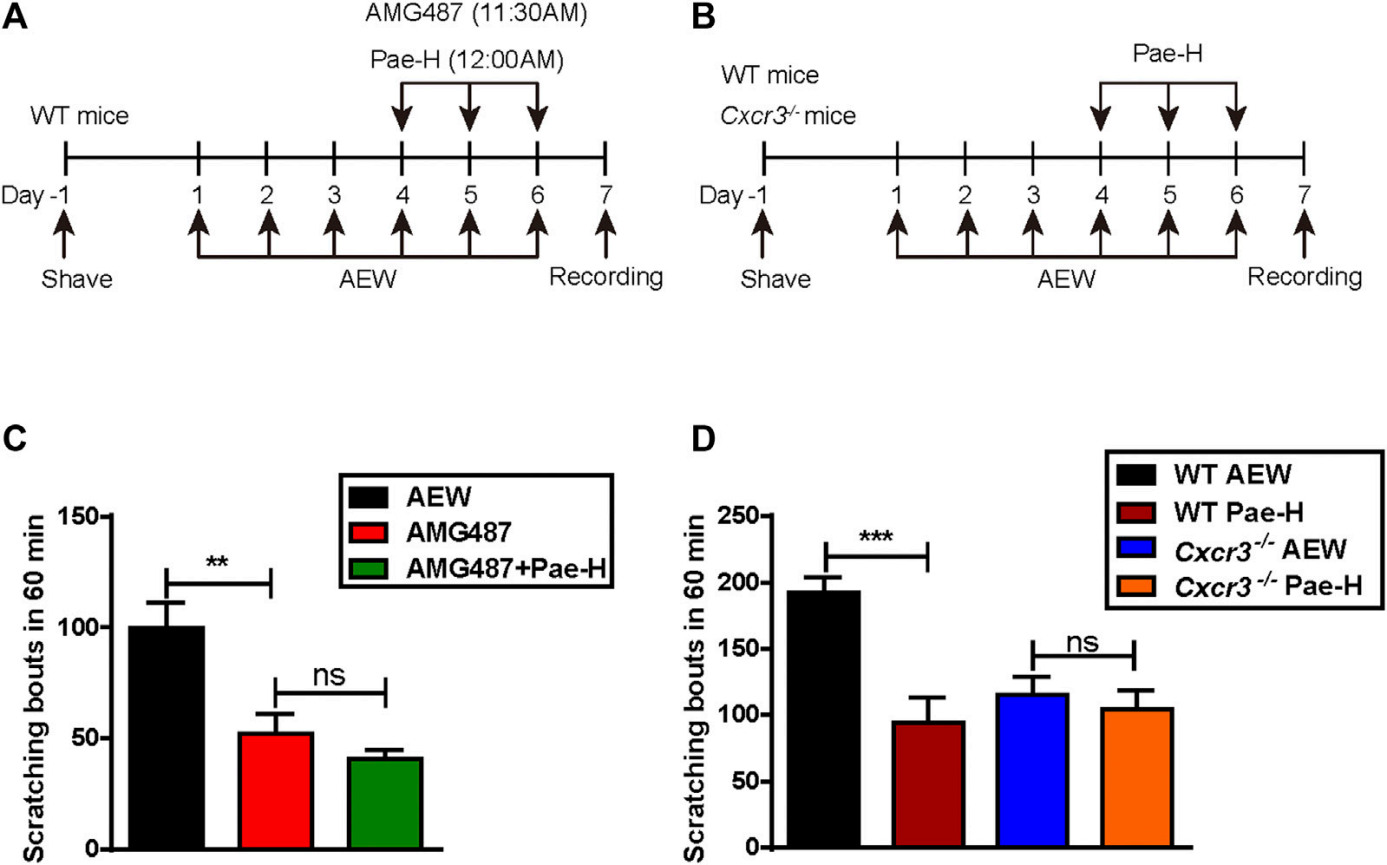
The effects of paeonol on chronic itch in AMG487 pre-treated WT and *Cxcr3* ^*−/−*^ mice. **(A)** Schematic protocol of CXCR3 inhibition and induction of AEW-induced dry skin model. **(B)** Schematic protocol of AEW-induced dry skin model in *Cxcr3* ^*−/−*^ mice. **(C)** The effects of paeonol on AEW-induced spontaneous scratching in WT mice pre-treated with AMG487. **(D)** The effects of paeonol on AEW-induced spontaneous scratching in *Cxcr3* ^*−/−*^ mice (*n* = 7–9 each group). Data were expressed as mean ± SEM. ^∗∗^ *p* < 0.01, ^∗∗∗^ *p* < 0.001, compared to the WT-AEW model group.

Then, we observed the effects of paeonol on the scratching bouts of AEW mice pre-treated with a single intraperitoneal injection of CXCR3 antagonist AMG487. The data of itching behavior indicated that paeonol failed to relieve the AEW-stimulated itch when CXCR3 was pharmacologically inhibited (Figures 7A–C). Similarly, though paeonol significantly impaired the scratching frequency in AEW-induced C57BL/6J mice, it was almost ineffective in *Cxcr3* ^*−/−*^ mice stimulated by AEW (Figures 7B–D), demonstrating that spinal CXCR3 was indispensable for the anti-pruritic activity of paeonol in AEW-induced dry skin.

### The Suppressive Effect of Paeonol on Astrocytic Activation Was Abolished in *Cxcr3* ^*−/−*^ Mice

**FIGURE 8:**
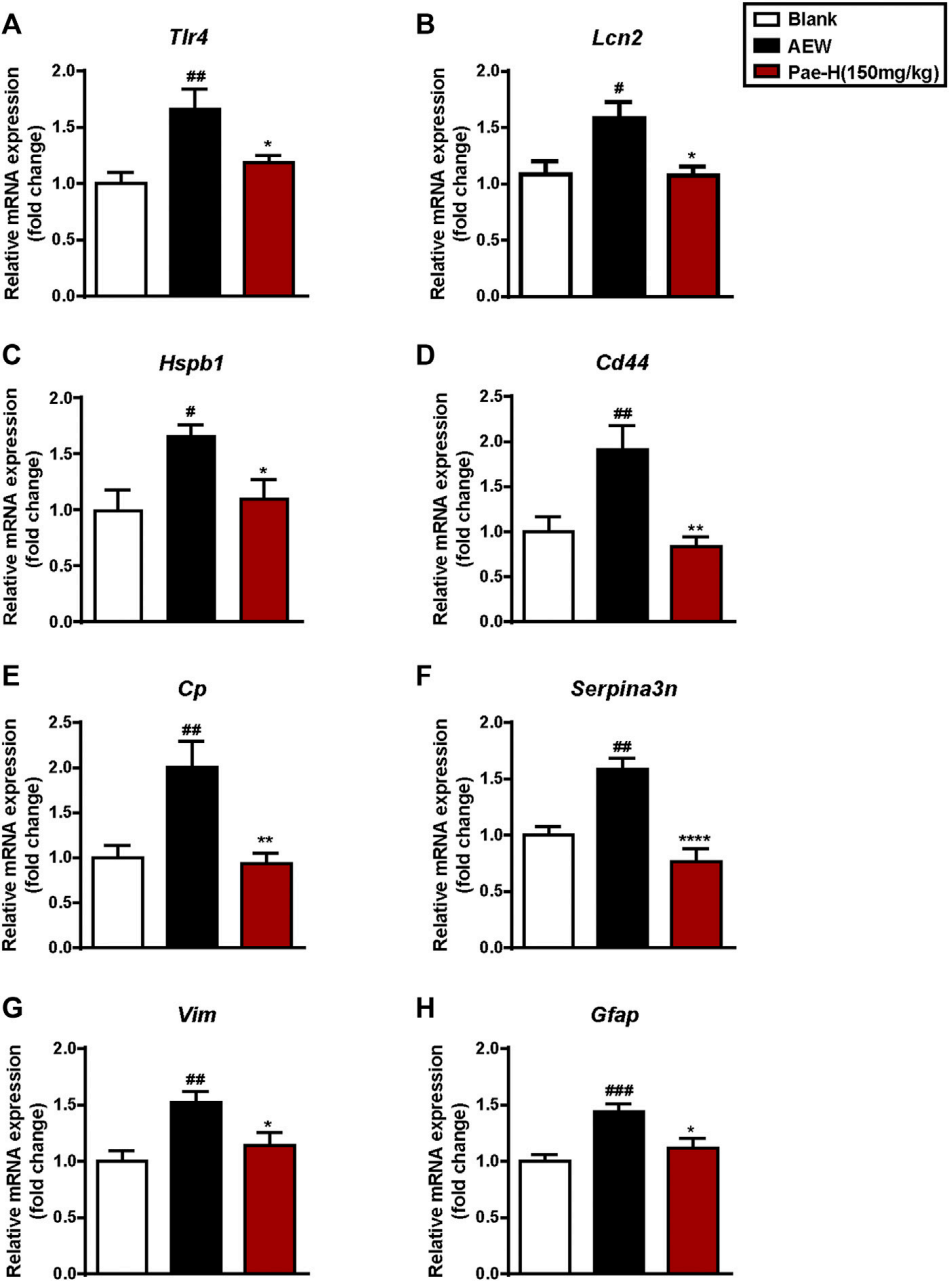
Paeonol suppressed the expression of astrocyte activity-dependent genes in the spinal cord of AEW mice. **(A–H)** Administration of paeonol inhibited the mRNA levels of genes related to reactive astrocytes (*n* = 5–9 each group). Data were expressed as mean ± SEM. ^#^ *p* < 0.05, ^##^ *p* < 0.01, ^###^ *p* < 0.001, compared to the blank group; ^∗^ *p* < 0.05, ^∗∗^ *p* < 0.01, ^∗∗∗^ *p* < 0.001, compared to the AEW model group.

**FIGURE 9:**
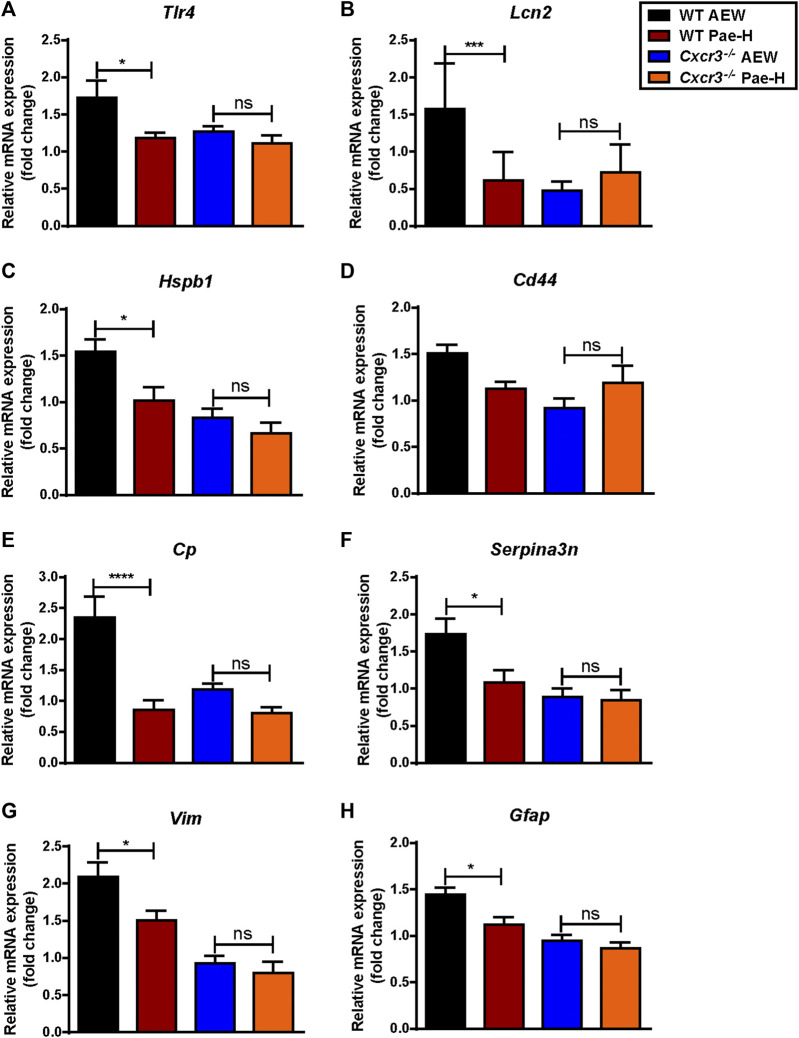
The effects of paeonol on astrocyte activity-dependent genes in WT mice and *Cxcr3* ^*−/−*^ mice. **(A–H)** The effects of paeonol on mRNA levels of genes related to reactive astrocytes in WT and *Cxcr3* ^*−/−*^ mice (*n* = 5–9 each group). Data were expressed as mean ± SEM. ^∗^ *p* < 0.05, ^∗∗^ *p* < 0.01, ^∗∗∗^ *p* < 0.001, ^∗∗∗^ *p* < 0.0001, compared to the WT-AEW model group.

Reactive astrocytes in the SDH have been reported in models of dry skin (Green and Dong, 2015; Liu et al., 2016; Tsuda, 2017; Tsuda, 2018) and activated astrocytes may produce CXCR10 to act on neurons through CXCR3 via a paracrine signaling (Jiang et al., 2017; Doron et al., 2019; Petrisko et al., 2020). To explore whether paeonol affects the activation of astrocytes in AEW model, we tested the mRNA levels of toll-like receptor 4 (*Tlr4*) (Liu et al., 2016) (Figure 8A), lipocalin 2 (*Lcn2*) (Shiratori-Hayashi et al., 2021) (Figure 8B) and other genes in the spinal cord related to reactive astrocytes (Liddelow et al., 2017) (Figures 8C–H), including heat shock protein 1 (*Hspb1*), CD44 antigen (*Cd44*), ceruloplasmin (*Cp*), serine/cysteine peptidase inhibitor, clade A, member 3N (*Serpina3n*), vimentin (*Vim*) and glial fibrillary acidic protein (*Gfap*), and found that their expression were significantly decreased by paeonol treatment as expected. Conversely, paeonol failed to unregulated the levels of these genes in AEW-induced *Cxcr3* ^*−/−*^ mice (Figures 9A–H). Therefore, paeonol was able to inhibit the astrocytic activation of AEW mice driven by CXCR3, suggesting that targeting spinal CXCR3 is a potential approach for treating the chronic itch with activated astrocytes.

## Discussion

Nowadays, increasing attention has been paid to the anti-dermatitis activities of paeonol. For example, paeonol inhibits the maturation and activation of DCs by reducing MyD88 and TLR8 proteins in the TLR7/8 signalling pathway, and ultimately alleviates psoriasis-like skin lesions in BALB/c mice (Meng et al., 2017). For UV-induced skin disorders, paeonol ameliorates SUV-induced skin inflammation by inhibiting the increase of TOPK, the phosphorylation of p38, JNKs and H2AX, and the secretion of IL-6 and TNF-α in Babl/c mouse. Paeonol also attenuates UVB-induced matrix metalloproteinase-1 production and promotes procollagen type I in hairless mice to protect skin from UVB-induced photoaging (Xue et al., 2017; Sun et al., 2018). In addition, paeonol improves the development of DNCB-induced AD in the BALB/c mice by reducing severity of the lesions, epidermal thickness and mast cell infiltration, accompanied by the reduction of immunoglobulin E and inflammatory cytokines, and regulation of the T helper (Th) cell subset (Th1/Th2) ratio (Meng et al., 2019). Nevertheless, these pharmacological studies mainly focused on anti-inflammatory activity of paeonol. Although the inhibitory effects of paeonol on scratching behavior and mast cell degranulation in the acute itch model induced by compound 48/80 (Lee et al., 2008) supported our findings in AEW-induced dry skin mice, the mechanism of its ability of antipruritus, especially for chronic itch, is poorly understood so far.

CXCR3 signaling has been reported involving in various human diseases, including chronic inflammation, immune dysfunction, cancer, metastasis, and pruritus (Van Raemdonck et al., 2015). CXCR3/CXCL10 axis contributes to the recruitment of inflammatory cells and the production of cytokines in RA progression (Lee et al., 2017), chronic prostatitis (Hua et al., 2021), inflammatory bowel disease (Zhao et al., 2017), etc. In addtion to its traditional functions in inflammation and immunity as we known, the potential role of CXCR3 in chronic dermatitis on itch sensation has attracted growing attention in recent years. AEW treatment induced the elevated levels of CXCR3 and CXCL10 in the spinal cord, and *Cxcr3* ^*−/−*^ mice showed reduced scratching behavior than that in control mice (Jing et al., 2018). Consistent with previous studies, we investigated that the expression of CXCR3 in the spinal cord of AEW mice was significantly elevated along the period. Treatment of paeonol effectively reversed the expression of spinal CXCR3 at both mRNA and protein levels. Though the expression of spinal *Cxcl10* in AEW-induced mice did not increase as previously reported, it was reduced by paeonol significantly as well, supporting the hypothesis that paeonol exerted as an anti-pruritic agent in the treatment of dry skin via inhibiting spinal CXCR3-mediated signalling. In addtion to *Cxcr3/Cxcl10* pair, it’s worth mentioning that paeonol also significantly down-regulated the mRNA levels of spinal *Ccl6*, *Ccl9* and *Ccl17*. However, there is little evidence of their exact roles in itch sensation.

Central circuit mechanisms that contribute to the sensation of itch, including the crucial role of astrocytes in maintaining the development and pathogenesis of chronic itch, has attracted widespread attention (Green and Dong, 2015; Chen and Sun, 2020). Recent studies have proved that the repetitive scratching behaviors in chronic itch models require the contribution of astrocytic molecules including the transcription factor signal transducer and activator of transcription 3 (STAT3) and the receptor toll-like receptor 4 (TLR4) (Tsuda, 2018). Behavioral evidence using NC/Nga mice suggested that STAT3-dependent spinal cord central sensitization occurs via amplification of GRP signalling under chronic itch conditions (Shiratori-Hayashi et al., 2015). LCN2 upregulated in STAT3-dependent reactive astrocytes in the SDH amplifies GRP signalling and contributes to the progression and maintenance of chronic itch (Shiratori-Hayashi et al., 2015; Shiratori-Hayashi and Tsuda, 2020; Shiratori-Hayashi et al., 2021). Spinal TLR4 is necessary for AEW-induced chronic itch in the cheek model. AEW induces persistent upregulation of *Tlr4* mRNA in GFAP-expressing astrocytes and TLR4-dependent astrogliosis (GFAP upregulation) in the spinal dorsal horn (Tsuda, 2018). In addition, reactive astrogliosis consists of a rapid, but quickly weakened, induction of gene expression after injury, including *Cp* (Ryan et al., 2018; Wu et al., 2018), *Vim* (Qian et al., 2015; Smith et al., 2018; Adolf et al., 2019) and *Serpina3n* (Zamanian et al., 2012; Domowicz et al., 2021; Ji et al., 2021). In our study, their expression responds to AEW stimulation increased significantly.

In addition, chemokines expressed in the central nervous system also participate in the pathogenesis of pain and itch via neuron-glia interaction in the spinal cord. CXCL13 is highly upregulated in spinal neurons after spinal nerve ligation and induces spinal astrocyte activation via receptor CXCR5 (Zhang et al., 2017; Liu S. et al., 2019). Absence of the chemokine receptor CXCR3 inhibited both astrocytic activation and chronic itch in AEW-induced dry skin model mice (Jing et al., 2018; Shiratori-Hayashi and Tsuda, 2020). In this research, the expression of astrocyte activation-dependent genes (*e.g., Tlr4*, *Lcn2*, *Hspb1* and *Cd44*) were significantly decreased by paeonol administration (Figure 4), proving that paeonol could inhibit AEW-induced activation of astrocytes in the spinal cord. However, the inhibitory effects of paeonol on these genes was abolished in CXCR3-deficient mice (Figure 5), supporting that paeonol relieved the pruritus of dry skin mice by inhibiting the spinal astrocytic activation driven by CXCR3. In fact, in recent years, the research field has made substantial progress in understanding how astrocytes at the spinal cord level participate in the regulation of chronic pain and pruritus through neuron-glia and glial-glia interactions. In addition to the effects of cytokines and chemokines in causing skin itching in the periphery, they may also participate in the above-mentioned interactions through the activation of astrocytes, causing chronic itching. It has been clearly showed that the mediators produced by astrocytes such as cytokines and chemokines are powerful neuromodulators that can regulate the itch circuit and cause central sensitization. For further studies, we plan to focus on the exact process of neuronal CXCR3 mediates the astrogliosis in the SDH, and the role of paeonol plays in it.

In conclusion, for the first time we investigated that paeonol could ameliorate both the chronic itch and skin inflammation of AEW-induced dry skin in mice. It exerted good anti-pruritic effect through suppressing the astrocyte activation which was driven by CXCR3 in the spinal cord. Thus, searching for new natural compounds that target spinal CXCR3 signaling may provide novel therapeutic potential for the treatment of dry skin disease.

## Data Availability

The original contributions presented in the study are included in the article/supplementary material, further inquiries can be directed to the corresponding authors.
